# Reasons for modern contraceptives choice and long-acting reversible contraceptives early removal in Amhara Region, Northwest Ethiopia; qualitative approach

**DOI:** 10.1186/s12905-023-02375-3

**Published:** 2023-05-19

**Authors:** Kiros Terefe Gashaye, Keflie Yohannes Gebresilassie, Belayneh Ayanaw Kassie, Chernet Baye Zenebe, Zelalem Mengistu, Solomon Emyu Ferede, Zewudu Andualem, Mehari W./Mariam Merid, Asefa Adimasu Taddese, Mikyas Abera

**Affiliations:** 1 Department of Obstetrics and Gynecology, College of Medicine and Health Sciences, University of Gondar, Gondar, Ethiopia; 2 Midwifery Directorate, College of Medicine and Health Sciences, University of Gondar, Gondar, Ethiopia; 3 Department of RFPH, School of Public Health, College of Health Sciences, Addis Ababa University, Addis Ababa, Ethiopia; 4 Department of Environmental and Occupational Health and Safety, Institute of Public Health, Collège of Medicine and Health Sciences, University of Gondar, Gondar, Ethiopia; 5 Department of Epidemiology and Biostatistics, College of Medicine and Health Sciences, University of Gondar, Gondar, Ethiopia; 6 PhD Student, Department of Health Informatics /Biostatistics/, Institute of Public Health, College of Medicine and Health Sciences, University of Gondar, Gondar, Ethiopia; 7 Department of Sociology, College of Social Science and Humanities, University of Gondar, Gondar, Ethiopia

**Keywords:** Modern contraceptives, Early removal, Qualitative, Choice, Ethiopia

## Abstract

**Background:**

Women use modern contraceptive methods, mainly either to limit or space pregnancy and both are not identical in their choices. One method may not best fit an individual’s need irrespective of the time of spacing. Cognizant of this, the context with which women base in choice of contraceptives, their lived experiences in using, and factors for early removal/ discontinuation of long-acting reversible contraceptives (LARCs) are not much investigated in the study setting and our study aimed to bridge the gap through exploring the underlying reasons.

**Method:**

A phenomenological study design was used to explore sampled women’s reasons and experiences. Reproductive-aged women (15–49 years) who removed long-acting methods in the past 6 months were included. A criterion sampling approach was employed to recruit study participants. Data was collected using an interview guide for in-depth (IDIs) and key informant interviews and were tape-recorded with interviewees' consent. Audio data were transcribed verbatim and translated into English. The data was first saved in plain text format and imported into Atlas.ti 7.0 software to facilitate coding and categorizing. The content analysis method was used to classify, organize data, and interpret the qualitative data according to key categories.

**Results:**

Several misconceptions about contraceptives (e.g., implants are not appropriate for daily laborers, women who use contraceptives (such as injectables) can only bear girl-child, etc.) were reported by clients and health providers. These misconceptions might not have scientific merit but they are powerful enough to affect actual behaviors toward contraceptives, including early removal. The awareness, attitude, and use of contraceptives tend to be lower in rural areas. For premature removal of LARCs, side effects, and heavy menstrual bleeding, was the most commonly identified reason. The IUCD is the least preferred method and users said it is not comfortable during sex.

**Conclusion and recommendation:**

Our study found different reasons and misconceptions for modern contraceptive methods’ non-use and discontinuation. Standardized counseling approaches like the REDI (Rapport Building, Exploration, Decision Making, and Implementation) framework should be implemented in the country consistently. Some of the concrete providers’ conceptions should be well-studied considering contextual factors to bring scientific evidence.

**Supplementary Information:**

The online version contains supplementary material available at 10.1186/s12905-023-02375-3.

## Introduction

From the scientific evidence, women use modern contraceptive methods mainly for two reasons as far as reproduction is concerned i.e., to limit or space pregnancy. But both spacers and limiters are not identical in terms of modern contraceptive use [1–5]. About 41% of reproductive-age women are currently using modern contraceptive methods [6]. But they tend to prefer one contraceptive method over the other for different reasons [7]. In line with FP2020 commitments, Ethiopia has planned to reach a contraceptive prevalence rate of 55%. This would mean reaching an additional 6.2 million women and adolescent girls with family planning services by 2020 (MOH 2015) [6]. According to Mini-EDHS 2019 report, the most popular contraceptive methods are injectable (27%), followed by implants (9%), and the pill and the IUD (2%) each [8].

Although the availability and utilization of LARCs have improved over the years, early discontinuation is emerging as a concern for public health policy and practice. Abdominal cramping, amenorrhea, and changes in bleeding patterns are commonly reported as reasons for removal [9–11]. However, a bundle of bottlenecks was expected to hamper the reaching of this goal. Several studies have shown that socio-demographic factors such as age, religion, residence, education, ethnicity, media exposure to family planning, limited access to family planning methods, provider readiness, and poor-quality service provision can prevent these commitments from translating into real progress [12–18]. Thus, ensuring access to, availability, and quality of a range of family planning services is one of the priority areas in which significant investments are required by countries and donors to meet FP 2020 Sustainable Development Goals [19]. Despite there have been a lot of efforts to identify factors affecting modern contraceptive utilization with quantitative studies, there is a paucity of evidence on the exploration of the lived experience, deep feelings and values of mothers on modern contraceptive method utilization in Ethiopia in general and study area (Northwest Ethiopia) in particular.

Shreds of evidence that will come out directly from end users will be very important information for policymakers, health planners, and family planning providers to modify and plan activities pertaining to family planning services in accordance with community interest and context while maintaining scientific evidence. Therefore, this project is aimed to accelerate contraceptive use focusing on the impediments to large-scale uptake of family planning services from different perspectives in the region. The General objective of this study is to explore the reasons for contraceptive choice, early removal of long-acting reversible contraceptive methods and to probe lived experience of women among modern contraceptive users in the Amhara Region, Northwest Ethiopia.

## Methods and materials

### Study setting/ area and design

The study was done in the selected health facilities found in Gondar, Woreta, Bahir Dar, Debre Markos, Debre Tabor, Shewa Robit, Woldia, Dessie, and Debre-Berhan cities from Sept to Nov 30/2020. The study sites were selected due to the presence of a high number of LARC users and to take representative samples from the region.

An institution-based qualitative exploratory study using a phenomenological approach was conducted. It is used to describe the reasons why women choose modern contraceptives, to explore the lived experience of women who utilized modern contraceptive methods for more than six months and the reasons why women discontinue LARC prematurely. It is a systematic and subjective approach to highlight and explain lived experiences and to further give them meaning. Understanding lived experiences marks phenomenology as a philosophy as well as a method. In this process, the researcher works to take the experiences of participants on the participants’ terms.

### Study population

All women in the reproductive age group came for contraception services in the selected health facilities during the data collection period. Women who have been on contraceptive methods for a minimum of six months were asked for their lived experience. Health Extension workers, providers, and health Managers who are actively working in the selected health facilities and community were also the study subjects of this study.

### Sample size determination and sampling techniques

A purposive sampling technique was employed to select 50 samples (8 healthcare providers, and 42 clients). Data was collected through In-Depth Interviews (IDI) and Key Informant Interviews (KII), using an interview guide in Amharic (local) language, consisting of questions (*on socio-demographic variables, client’s awareness of FP (particularly about LARC, the reasons for early removal and post-removal experience*) that are not rigidly adhered to, but served for a structured conversation and to ensure all topics covered. Free flow of information was encouraged through probing.

IDI was done until saturation is attained for (a) women who currently come to use (*upon exit from the facility on why they choose the method*); (b) who have been on modern contraceptives for a minimum of 6 months (*purposively selected from previous family planning registration logbooks and called to come to the health facility, get their voluntary consent and interviewed for their lived experience*) and (c) for early removal of LARCs (*before LARC removal service*).

For KII, healthcare providers and Health Managers were used as key informants to explore challenges of contraceptive utilization, reasons for contraceptive choice, and methods early discontinuation.

### Data management and analysis

The IDI was taped and noted (key information). Audio data were transcribed verbatim into word files and translated to English; key terms were reviewed in Amharic language and translated to ensure a degree of standardization. Final transcripts were compared against notes taken to ensure quality. Before the analysis, the text was read several times to be familiar with the data. First, the data is saved in text format and imported into Atlas.ti 7.0 software to facilitate coding and categorizing. It was independently coded by two investigators. The various codes were compared based on differences and similarities and sorted into categories. Finally, based on content analysis, the underlying meaning that which is the latent content of the text was formulated under each of the categories. The findings encompass direct quotes of women and were narrated without editing the grammar to avoid loss of its meaning. Quotes that best described the categories and frequently mentioned ideas were chosen from several groups.

## Results

### Summary profile of study participants – clients and providers

A total of 41 contraceptive users and 9 health workers (one in each study locality, except Dessie where two health providers were interviewed) were drawn from selected urban areas in the Amhara region. All the clients interviewed were women in the age range of 16–38 years old, while the majority (86%) were Orthodox Christians. The overwhelming majority (86%) were married at the time of the study. Regarding the cases, the majority (55%) of interviewees were Long-Acting Reversible Users. (Table 1).

**Table 1:** Sociodemographic characteristics of the study participants

Characteristics	Number (Percentage)
**Sex**	
Female	48 (96%)
Male	2 (4%)
**Religion**	
Orthodox Christians	43 (86%)
Muslim	7 (14%)
**Marital Status**	
Married	43 (86%)
Divorced	6 (12%)
Never Married	1 (2%)
**Type of cases selected**	
Contraceptive choice	7 (14%)
Early removal of LARC	12 (24%)
Currently LARC-User	22 (44%)
Health workers	9 (18%)

Regarding the health workers interviewed, eight (88.9%) were women (and one man), and they were aged 23–45 years old (See Annex 1).

### Reasons of clients for choosing or not choosing a contraceptive method

Women’s contraceptive choices and their plans depend on numerous factors including their procreation plan, perceived health status, and comfort levels with specific contraceptives. Several reasons were mentioned for choosing a certain contraceptive method. Contraceptives related *Side effects* are frequently cited reasons for women opting to use current methods. A 25-year-old Depo-Provera user (Woldia) and well-knowledgeable about the different contraceptive methods reports, “I have used injectables for nine months. But I do not feel comfortable when using it as it reduces my breast milk. It also reduced my weight from 65 to 53 kg. Health professionals recommended implants. But I do not prefer it because my sister used it and caused heavy menses.”

*Health workers* were also cited as the reason for choosing a contraceptive method for the women following side effects. For choosing an implant, a 24-year-old mother of two (Debre Birhan) mentioned that “Injectable is prohibited…. They [health providers] said implant is better than injectable. Injectable dries breast milk. It is not recommended for lactating mothers as she [health provider] said. Therefore, my choice is implant for my child…. The providers tell the truth, and then we choose the best. I chose implant on their advice. The decision was mine.” Another 26-year-old never-married implant user reports her method-shift experience, “It [Depo-Provera] was just irritating me and sometimes I got excessive menses. When I consulted with health professionals, they advised me to stop it and change to implant.” Discussing health workers’ counseling services, a 22-implant user adds, “Yes. They advised me and I weighed its benefits and side effects and found it [implant] to be balanced… [and] better than injectable.”

*A woman’s clinical condition* such as breastfed determines which contraceptive methods to use as a mother of one (22-year-old housewife) implant-user says “I was breastfeeding and the implant is preferable for women who are breastfeeding due to its hormones. That is why I chose it…. People say injectable results in drying of breast milk.” *Experimenting among the different methods* was reasoned for other women– as a 27- year-old mother of two injectable-user (Debre Markos) explains, “Because, I never tried it before. I see all the other LARCs are not appropriate for me.” The experimentation involves shifting from one method to another.

*Women’s fears of medical procedures*: were also reported by Interviewees (both users and health workers) in administering LARCs – than cultural or religious factors – to explain why they do not choose LARCs. For instance, a 25-year-old mother of two who uses implant (Woreta) says, “They (people) are afraid about insertion [of methods] into their arm. But it is comfortable when compared to injectable." A female health provider in Bahir Dar (28 years old with 3 years of experience) added that it is “due to fear, even if we give them detailed information about its side effects and advantages, they said no for that day. They choose injectable and promise to use implants [in the future] or after consulting with their husbands or partners.”

*Community views and attitudes towards contraceptives*- affect the use or non-use of contraceptives. This is especially the case in rural areas, a 25-year-old mother of one who uses Depo-Provera (Woldia) explains,

I was born in a rural area, where most people are happy to use contraceptives. People have no negative attitude regarding contraceptives… Some people said that it might cause infertility, skin darkening, and coughing; that it might [affect the body] long after discontinuation; that women who use contraception give birth to girl-child; that women who use contraceptives cannot bear a boy-child. But I didn't use it before I gave birth to two girls... Most of the time, the injectable is associated with giving birth to girls.

The challenges and perceptions are strong in the case of IUDs as women explained. A 22-year-old implant user says, “They are afraid of it. They believe that it causes a problem…. I didn’t know people who use it [either]. Mostly, they use injectables and people don’t use those inserted inside the arm, inside the uterus, etc.” The side effects and misconceptions surrounding its use have been raised as reasons for the poor utilization of IUDs. A 30-year-old mother of four who uses an implant (Gondar) report, for instance, “IUD causes heartburn, increases body temperature, and weight gain….” Similarly, 23-year-old daily labor who uses an implant (Tseda, Gondar) says, “I never used IUD. People say it affects the uterus, it can’t retain pregnancy, and causes cancer.” On the other hand, the method is best chosen by another woman also as a 28-year-old mother of one implant-use says, “Some people say implant causes infertility and it affects body weight. They take a loop (IUD) as best for its [fewer] side-effects….”

### Experience of LARCs users (Implant and IUD)

A lot of LARC experiences were discussed by women. There was a method shift from short-acting to long-acting and vice versa. Woman’s previous experience with contraceptive uses influence their decision for current use. A 35-year-old Depo-Provera user explains her implant experience (already discontinued) as: “I used to have heavy menses, which flows for 15 days straight.… [This started] 2 years after insertion. I consulted providers and they gave me pills taken orally [to correct for the heavy bleeding]. But there was no change…. and I had to remove it.”

A 27-year-old married woman (Gondar) was using short-acting contraceptive methods for the past 10 years and shifts at different times due to the side effects. She finally inserted implants and explains her continued plan to use it “I’ll use it until I want a child or it’s time for replacing the current one. In the future, I might use IUD if it is better. But now, I will continue using implants as I have no plan to get pregnant for the next 6 years.”

In Contrast, some other woman also reaches a decision not to use any of the methods. A 26-year-old mother of two (Debre Markos) who removed the implant on the day of the interview says, “I’m not comfortable with contraceptives, and [they] hurt my body. I decided not to use any. I warned and told my husband too [i.e., to abstain from any sexual activities].” In Gondar, a 38-year-old mother of five explains why she intends to shift from one contraceptive to another: “I don’t think I will use [implant], because it causes problems on me. [I think] it will be good if I simply use COCs and Depo.”

### Reasons for early removal of LARCs

Some women could properly use LARCs for their intended duration, while others discontinue them prematurely due to age, prior contraceptive experience, and side effects such as heavy menses. A 38-year-old health provider (Debre-Birhan) with 12 years of work experience identified clients’ age as one of the factors affecting the decision to remove LARCs early: “[Mostly] older clients use it for the whole duration. But adolescent clients usually come for early removal when they get separated from their boyfriends.”

*Side Effects-* Broadly speaking, nonetheless, there are “clients who discontinue LARCs for reasons including irregular and excess menses, which is especially associated with Implanon, or when they want to have a child.” A health provider adds, “Clients provide several reasons to remove LARCs early including arm exhaustion, menstrual irregularity, and behavior change (‘it made me angry’) and weight loss.” Women could also experience complications from these side effects as a 30-years-old health provider explains,

Most of them [clients] discontinued it [Implant] due to severe bleeding. All contraceptives that I know cause bleeding. In my opinion, the impact of each type of contraceptive especially implants should be studied. I know a mother who became anemic and got admitted to the hospital for a few days for blood transfusion and oxygenation.

These side effects mentioned by women should not be underestimated as it exacerbates pre-existing health conditions, and critically affects their personal and socioeconomic activities A 29-years-old implant user (Gondar) says, “I was using an implant and I had to remove it after four months as my sugar level increased. My sugar level had increased before I get pregnant with my daughter. But during pregnancy, it subsided, but after I gave birth and started using an implant, it again started to increase.” Another 37-year-old married woman (Dessie) says, “If you have frequent menses, it is difficult to have sexual intercourse and you may separate from your partner. I know a mother who discontinues it because of this.” A married street-vendor woman, who removed LARCs after three weeks of insertion (Gondar), explained her experiences:

My hand feels numb and I was unable to work. My menses were excessive. It continued for three weeks starting from the 3^rd^ or 4^th^ day of the implant…. I could tolerate the bleeding and they have also told me it has medications to correct it. But my reason for removal is my frail left hand. I’m unable to work, which requires me to carry a basket full of bananas as I street-vend. I do not want to remove it but it doesn’t match the nature of my work. I haven't been able to work for the past three weeks as the bleeding is heavy and moving about is difficult.

Health workers also agree with the side effects of contraceptives on women’s situations. A health provider in Debre Markos reported that “I can say more than 90% of women discontinue LARCs prematurely due to bleeding.” This is particularly challenging for Muslim clients, she adds, since it makes religious observance, such as praying very difficult for them and leads them to request early removal services. However, they are also reluctant to give removal services as a 22-year-old married woman (Woreta) who had recently removed Implanon says, “Many women complain the three- and five-year contraceptives are not comfortable. They cause a lot of bleeding, change behavior, and lead to significant weight loss. Moreover, the providers are not interested to remove the implant.”

Discussing timelines for LARCs removal among clients in and around Woreta, a 29-year-old health provider reports, “Most [LARCs] users remove it within two to three years after insertion. But some women remove it within a year [for reasons including] divorce, getting married, family pressure to bore child, and excessive menses.”

Women may use or not use other contraceptives after discontinuing LARC, which depends on the reasons for discontinuation. Most shift to injectables as health workers explains. A 28-year-old female health provider (Bahir Dar) reports, “They mostly shift to injectable after removing LARCs. If they are separated/divorced from their husband, [however] they remove it and avoid contraceptives altogether.” Another 29-years-old male health provider (Woreta) adds, “Most of them use the three-month Depo-Provera and sometimes Combined Oral Contraceptive (COC) pills.”

## Discussion

In this study, clients choosing or not choosing contraceptives have been affected by several factors including the contraceptive side effects, the social interaction (spouses, families, and friends in choosing/not choosing contraceptives), contraceptive-related characteristics, and clients’ interaction with the contraceptive providers. Choosing a particular method depends on its effectiveness and ease of availability [20]. Similar to the findings of the current study, young adults prefer IUD for its lower hormone dose [21], and the ring than IUD for ease of its removal when not needed [22]. On the other hand, the main reasons for not choosing LARCs were their side effects and displacement that may result in an unwanted pregnancy. These findings were supported by previous studies on the subject [7].

Regarding the reason for early removal of LARCs; heavy menstrual bleeding, headache, weight loss, behavioral change [aggressiveness], and desire to have a child were commonly identified reasons, while heavy menstrual bleeding was the most common reason. The findings of this study are consistent with other previous studies [1, 23–28]. The possible explanation might be due to the genetic makeup of individuals, the types of work they undergo in their daily activities, community rumors on family planning, and the lack of proper pre-insertion counseling. This is supported by studies done in Ethiopia [1, 29]. Most study participants who use injectables were associated with birth delay and delivering only female children after using injectables and terminated to bring forth. Further investigation should be required to confirm the evidence and awareness creation required for users using mainstream media. We also found that some IUCD users faced unintentional pregnancy and they didn’t prefer to use it, especially those who lived in a rural community. This study finding is supported by a study conducted in Ethiopia [30]. Overall, family planning coverage in Ethiopia; injectables (23%), implants (8%), and IUDs (2%) [1]. The lowest coverage is IUCD this might be due to clients may not feel comfortable showing their uterus, it is a shame to show the uterus in Ethiopian culture, and the users said it is not comfortable during sex.

Finally, this study found that client-provider interaction during the counseling section is one of the factors that affect the choice of contraceptives. Some participants indicated that providers influenced their contraceptive choices – persuading them to choose particularly implants. This is a widespread problem as documented in the literature. Clients may harbor implicit bias towards their providers whereby they feel pressured to choose IUD when they were told more about IUD than other methods [31]. This is contrary to the standard procedure where the provider’s role should be provision of the required information and let the client decide on her own [32, 33].

## Conclusion and recommendation

Generally, it was possible to identify two very important intervention areas that had a significant influence on contraceptive choice. First community misconceptions about contraceptives and their side effects and second from the providers’ side in which some providers were not able to provide the required information and inform the clients about all the available contraceptives during the counseling sessions. In addition, individual clients’ health conditions and reproductive health plans affect once decision of choosing contraceptive methods. Thus, client-centered counseling approaches should be followed to improve service utilization using the different method mix. Researchers who wish to undergo similar studies may include focus group discussions to strengthen the quality of the research and if possible, they should be done ingredient characterization of different contraceptive methods to know why most of clients complain heavy bleeding and irregularity of menses.

## Supplementary Information

**Additional file 1:** **Annex-1.** The Summary Profile of Study Participants – Clients And Providers In Amhara Region, 2020.

## Data Availability

Data will be available upon request from the corresponding author.
