# Supplementary material for: Reasons for modern contraceptives choice and long-acting reversible contraceptives early removal in Amhara Region, Northwest Ethiopia; qualitative approach
Source: BMC Womens Health. 2023 May 19;23:273. doi: 10.1186/s12905-023-02375-3 (PMC10199468; doi:10.1186/s12905-023-02375-3)
Supplement: Supplementary file 1 — Additional file 1: Annex-1. The Summary Profile of Study Participants – Clients And Providers In Amhara Region, 2020. [file 12905_2023_2375_MOESM1_ESM.doc]

**Annex-1** The Summary Profile of Study Participants – Clients And Providers In Amhara Region, 2020

| **Case no.** | **City** | **Age/sex (provider)** | **Marital status** | **Education** | **Occupation** | **No. of children/clients** | **Religion** | **Case profile** |
| --- | --- | --- | --- | --- | --- | --- | --- | --- |
| 37 | Bahir Dar | 30 | Divorced | Undisclosed | Daily-laborer | 2 | Orthodox Christian | LARC-user |
| 01 | Bahir Dar | 30 | Married | Uneducated | Housewife | 4 | Orthodox Christian | Depo-Provera |
| 03 | Debre Birhan | 23 | Married | Grade-10 | Housewife | 1 | Orthodox Christian | LARC-user |
| 04 | Debre Birhan | 28 (F) | Married | Diploma | Nurse | 1000 clients | Orthodox Christian | Health provider |
| 05 | Debre Birhan | 24 | Married | Grade-5 | Merchant | 2 | Orthodox Christian | LARC-user |
| 06 | Debre Birhan | 21 | Married | 10+3 | Student | 0 | Orthodox Christian | LARC-user |
| 07 | Debre Birhan | 28 | Married | Grade-10 | Student | 0 | Orthodox Christian | LARC-user |
| 08 | Debre Markos | 27 | Married | Grade-10 | Housewife | 2 | Orthodox Christian | Depo-Provera |
| 09 | Debre Markos | 32 | Married | Grade-10 | Housewife | 2 | Orthodox Christian | Depo-Provera |
| 10 | Debre Markos | 22 | Married | Grade-10 | Housewife | 1 | Orthodox Christian | LARC-user |
| 11 | Dessie | 18 | Married | Grade-10 | Housewife | 0 | Orthodox Christian | Depo-Provera |
| 14 | Dessie | 45 (F) | Married | BSc | Nurse | 1800 clients | Muslim | Health provider |
| 15 | Dessie | 26 | Married | Grade-8 | Housewife | 1 | Muslim | LARC-user |
| 16 | Dessie | 32 | Married | BSc | Nurse | 9000 clients | Muslim | Health provider |
| 17 | Dessie | 21 | Married | Grade-9 | Waitress | 0 | Muslim | LARC-user |
| 18 | Gondar | 32 | Married | Uneducated | Daily-laborer | 2 | Orthodox Christian | Depo-Provera |
| 19 | Gondar | 20 | Married | Grade-10 | Student | 0 | Orthodox Christian | LARC-user |
| 21 | Gondar | 27 | Married | Grade-10 | Merchant | 2 | Orthodox Christian | LARC-user |
| 22 | Gondar | 23 | Married | Grade-10 | Merchant | 1 | Orthodox Christian | LARC-user |
| 23 | Gondar | 30 | Married | Uneducated | Housewife | 4 | Orthodox Christian | LARC-user |
| 24 | Gondar | 38 | Married | Uneducated | Housewife | 5 | Orthodox Christian | Early removal |
| 25 | Shewa Robit | 30 (F) | Married | BSc | Nurse | 2 | Orthodox Christian | Health provider |
| 26 | Woldia | 28 | Married | Grade-10 | Housewife | 2 | Orthodox Christian | LARC-user |
| 27 | Woldia | 25 | Married | MSc | Lecturer | 1 | Orthodox Christian | Depo-Provera |
| 30 | Woldia | 26 | Married | Uneducated | Daily-laborer | 0 | Muslim | LACR-user |
| 31 | Woreta | 29 | Married | Uneducated | Daily-laborer | 1 | Orthodox Christian | Depo-Provera |
| 32 | Bahir Dar | 24 | Married | BA | Private-sector | 0 | Orthodox Christian | Early removal |
| 33 | Debre Birhan | 26 | Married | 10+3 | Accountant | 0 | Orthodox Christian | Early removal |
| 34 | Debre Markos | 25 | Married | BA | Secretary | 2 | Orthodox Christian | Early removal |
| 35 | Dessie | 28 | Married | 10+3 | Teacher | 1 | Muslim | Early removal |
| 36 | Dessie | 27 | Married | 10+2 | Merchant | 1 | Orthodox Christian | LARC-user |
| 38 | Gondar | 29 | Married | Grade-8 | Housewife | 2 | Orthodox Christian | Early removal |
| 39 | Gondar | 28 | Married | Grade-10 | Civil servant | 1 | Orthodox Christian | Early removal |
| 40 | Gondar | 18 | Married | Uneducated | Street vendor | 1 | Orthodox Christian | Early removal |
| 41 | Shewa Robit | 30 | Married | 10+3 | Housewife | 1 | Orthodox Christian | Early removal |
| 42 | Debre Markos | 26 | Married | Diploma | Civil servant | 2 | Orthodox Christian | Early removal |
| 43 | Woreta | 28 | Married | Grade-5 | Self-employed | 2 | Orthodox Christian | Early removal |
| 44 | Woreta | 22 | Married | 10+3 | Student | 0 | Orthodox Christian | Early removal |
| 45 | Woreta | 29 (M) | Married | BSc | Nurse | 1000 clients | Orthodox Christian | Health provider |
| 46 | Woreta | 25 | Married | Grade-10 | Housewife | 2 | Orthodox Christian | LARC-user |
| 47 | Woreta | 27 | Married | Grade-10 | Housewife | 1 | Orthodox Christian | LARC-user |
| 48 | Bahir Dar | 28 (F) | Married | BSc | Nurse | 6300 clients | Orthodox Christian | Health provider |
| 49 | Bahir Dar | 28 | Married | Undisclosed | Housewife | 2 | Orthodox Christian | LARC-user |
| 50 | Debre Markos | 25 (F) | Married | Diploma | Nurse | 1200 clients | Orthodox Christian | Health provider |
| 02 | Debre Birhan | 19 | Never married | Grade-12 | Student | 0 | Orthodox Christian | LARC-user |
| 12 | Dessie | 26 | Never married | Uneducated | Daily-laborer | 0 | Orthodox Christian | LARC-user |
| 13 | Dessie | 16 | Never married | Grade-7 | Student | 0 | Muslim | LARC-user |
| 29 | Woldia | 25 | Never married | Uneducated | Merchant | 1 | Orthodox Christian | LARC-user |
| 20 | Gondar | 23(F) | Unmarried | Diploma | Midwife | 900-1000 clients | Orthodox Christian | Health provider |
| 28 | Woldia | 39(F) | Unmarried | BSc | Nurse | 1350-1800 clients | Orthodox Christian | Health provider |
